# Synthesis of SiC/SiO_2_ core–shell nanowires with good optical properties on Ni/SiO_2_/Si substrate via ferrocene pyrolysis at low temperature

**DOI:** 10.1038/s41598-020-80580-y

**Published:** 2021-01-08

**Authors:** Bo-Yu Chen, Chong-Chi Chi, Wen-Kuang Hsu, Hao Ouyang

**Affiliations:** Department of Materials Science and Engineering, National Tsing Hua University, Hsinchu, 30013 Taiwan, ROC

**Keywords:** Materials science, Materials for devices, Materials for optics, Nanoscale materials

## Abstract

In this study, the high-density SiC/SiO_2_ core–shell nanowires were synthesized on the nickel coated SiO_2_ (100 nm)/Si substrate by chemical vapor deposition (CVD) method with ferrocene precursor at temperature 1000 °C compared to previous studies (1300–1600 °C). The present work provides an efficient strategy for the production of SiC/SiO_2_ nanowires with uniform morphology and good optical properties, where the Ni layer plays important roles for this fabrication at low temperature which reduces the decomposition temperature of hydrocarbon gases and improves the growth quality of SiC nanowires. The as-synthesized SiC/SiO_2_ nanowires consist of single crystal 3C structures as well as 3C structures with defects along [111] direction. In the photoluminescence (PL) spectrum, the SiC/SiO_2_ core–shell nanowires revealed an obvious blueshift. The blueshift is due to the formation of nanoscale silicon carbide polytypism caused by the stacking faults in 3C–SiC and the nanoscale polytypism also caused the transition from indirect to direct bandgap which explains why the stacking faults percentage in SiC confirmed from X-ray diffraction (XRD) is 19%, but ultimately makes the strongest emission intensity. Finally, the PL characteristics are further improved by changing the diameter of the SiC nanowire and etching and an approximate model followed by the vapor–liquid–solid (VLS) mechanism was proposed to explain the possible growth mechanism of the SiC/SiO_2_ nanowires.

## Introduction

One-dimensional (1D) silicon carbide (SiC) nanomaterials such as nanowires, nanotubes, nanoneedle, nanobelts, and nanocables have attracted much attention due to their outstanding properties, such as high thermal conductivity, high mechanical strength, high electron mobility, chemical inertness, biocompatibility and wide band gap. Therefore, they can be used in microelectronics and optoelectronics devices, gas detection, field emission device, biomolecule-delivery vectors or intracellular electrodes, and high temperature nanoscale devices^1–4^.

For the application of blue and violet light emitters in displays, LEDs devices, SiC materials show a very stable luminescence and can be used in harsh environments and strict requirements due to the high temperature chemical and thermal stability^4,5^. Although the bulk SiC shows weak emission at room temperature because of its indirect band gap but this problem can be solved by diminishing the crystallite size to several or tens of nanometers^4,6^. However, there are still some challenges for SiC nanomaterial’s photoluminescence (PL) characteristics. The PL characteristics are observed to be quite variable, which strongly depends on the morphology (SiO_2_), size (diameter), structure (defects)^7–25^. Shena et al.^20^ synthesized the SiC/SiO_2_ nanowires with emission peak at 408 nm, the SiC/SiO_2_ nanowires synthesized by Zhang et al.^17^ shows emission peak at 438 nm and Shen et al. synthesized the SiC/SiO_2_ nanowires with emission peak at 401 nm^19^.

So far, several methods have been used to grow SiC nanowires. These synthesis methods include chemical vapor deposition (CVD)^12,17,23^, carbonthermal reduction^13,24^, vapor phase epitaxy^26^, arc-discharge^7^ and solid state reaction route^27^, etc. Among these the chemical vapor deposition (CVD) is the easiest and mostly used method because of the high possibility of large area growth and easy to control the shape and composition of the products^28,29^. However, conventional CVD process requires high deposition temperature because the hydrocarbon gas decomposition is an endothermic process^30,31^. In the absence of a catalyst, it requires a high operating temperature of about 1200 °C^30,31^. However, the operating temperature can be lowered, if a suitable metal catalyst is added (ex: Fe, Co, Ni)^29–32^. Besides, in order to grow SiC nanowires at lower temperature, many precursors have been tried, such as methyltrichlorosilane (MTS)^33,34^, polycarbosilane (PCS)^35^, polymethylsilane (PMS)^36^, ferrocene^37–41^ or mixed precursors^42,43^, etc. Among these, ferrocene is a cheaper and safer precursor and it has been demonstrated to be an efficient catalyst precursor for the synthesis of carbon nanotubes^44^, whereas it is seldom to be used for the synthesis of SiC/SiO_2_ nanowires. Niu and Wang synthesized 3C–SiC nanowires via CVD by evaporating ferrocene onto a Si wafer at 1550 °C^37^. Hu et al. synthesized SiC/SiO_2_ nanowires with a ferrocene-assisted chemical vapor reaction process at 1400 °C^38^. Li et al. synthesized SiC nanowires with ferrocene by a CVD route at 1500–1600 °C^39^.

For the ferrocene pyrolysis, it will be decomposed into the iron, hydrogen and hydrocarbon gas at temperature above 500 °C and the hydrocarbon gas is the source of carbon in silicon carbide^45^.

Although the Ferrocene has iron atom, decomposition efficiency of hydrocarbon gas is poor for growing CNT according to YD Lim et al. study^44^. For the hydrocarbon gas decomposition process, the catalyst activities are Ni > Co > Fe, the nickel has the best growth quality (growth rates and yield)^30,32,46^. Thus, the nickel thin film will be deposited on the substrate in advance to improve the quality of SiC growth in this study. Besides, it is also necessary to study the ferrocene as precursor to synthesize SiC nanowire at lower temperature to offer an energy-effective and cost-effective strategy.

In this study, the high-density SiC/SiO_2_ core–shell nanowires were synthesized on the nickel coated SiO_2_ (100 nm)/Si substrate by chemical vapor deposition (CVD) method at 1000 °C with ferrocene precursor. The CVD technology was used to control the shape and composition of the as-synthesized nanowires to improve PL properties which are not worse than the previous papers^7–25^.

## Experiment

Ni films were deposited on the SiO_2_ (100 nm)/Si substrate with the size 2.0 × 2.5 cm^2^ at room temperature by ion-beam deposition system under the base pressure of 8.0 × 10^–8^ Torr in the chamber. Before deposition, the substrate was cleaned by an ultrasonic cleaner for 20 min by using acetone, ethanol and DI water and then was dried in nitrogen flow. The Ni target (purity 99.99%) was pre-sputtered for 10 min to eliminate the contamination on the target surface before deposition. The beam voltage and beam current of the dc ion-source was set to 800 V/7.0 mA and the sputtering pressure is 7.2 × 10^–5^ Torr. The final thickness of Ni film was about 40 nm with deposition rate 0.03 nm/s. After the deposition of Ni, the sample was placed into the quartz tube furnace with a precursor—Ferrocene (C_5_H_5_)_2_Fe (purity 99.0%, 1200 mg), as shown in Fig. S1. The furnace is divided into three parts, where the first part was set to 400 °C, and the second and third parts were adjusted to 1000 °C respectively to grow nanowires. The precursor was placed 7 inches at the left side of the furnace and the sample was put on the second part of the furnace. Before heating, the vacuum in the quartz tube will be evacuated to 1.0 × 10^–2^ Torr, and then N_2_ gas (purity 99.999%) is injected into the quartz tube to 1 atm, and then the vacuum in the quartz tube will be evacuated to 1.0 × 10^–2^ Torr again, the above steps will be repeated 3 times to purify the atmosphere in the quartz tube. After that, it will take 3 h to slowly increase the temperature to the target temperature (400 °C, 1000 °C, 1000 °C) and then held for 1 h at a constant N_2_ flow of 160 SCCM to keep the pressure at 1 atm to grow nanowires. The quartz tube was naturally cooled to room temperature. The parameters of film deposition and CVD were summarized in Tables S1 and S2.

X-ray diffractometer [XRD (Rigaku-TTRAX III)] with the Cu target (λ = 1.5406 Å) was used to determine the crystalline phases. The scanning electron microscopy (SEM) (FESEM-8000) was used to analyze the surface morphology. The microstructure and composition of the sample were investigated by transmission electron microscopy (TEM) (JEOL ARM-200FTH) with Energy-dispersive X-ray spectroscopy (EDS) and simulation software JEMS (Java Electron Microscopy Simulation Software)^47^. For TEM observation, the as-synthesized SiC/SiO_2_ nanowires were dispersed in ethanol (purity 99.5%) by ultrasonication for 15 min, and then a droplet of solution was dropped onto a lacey carbon-coated copper grid (300 mesh). The detail procedure is shown in Fig. S2. The photoluminescence (PL) spectrum was performed at room temperature using a fluorescence spectrophotometer (PerkinElmer LS55) with the excitation wavelength of 325 nm.

## Results and discussion

**Figure 1:**
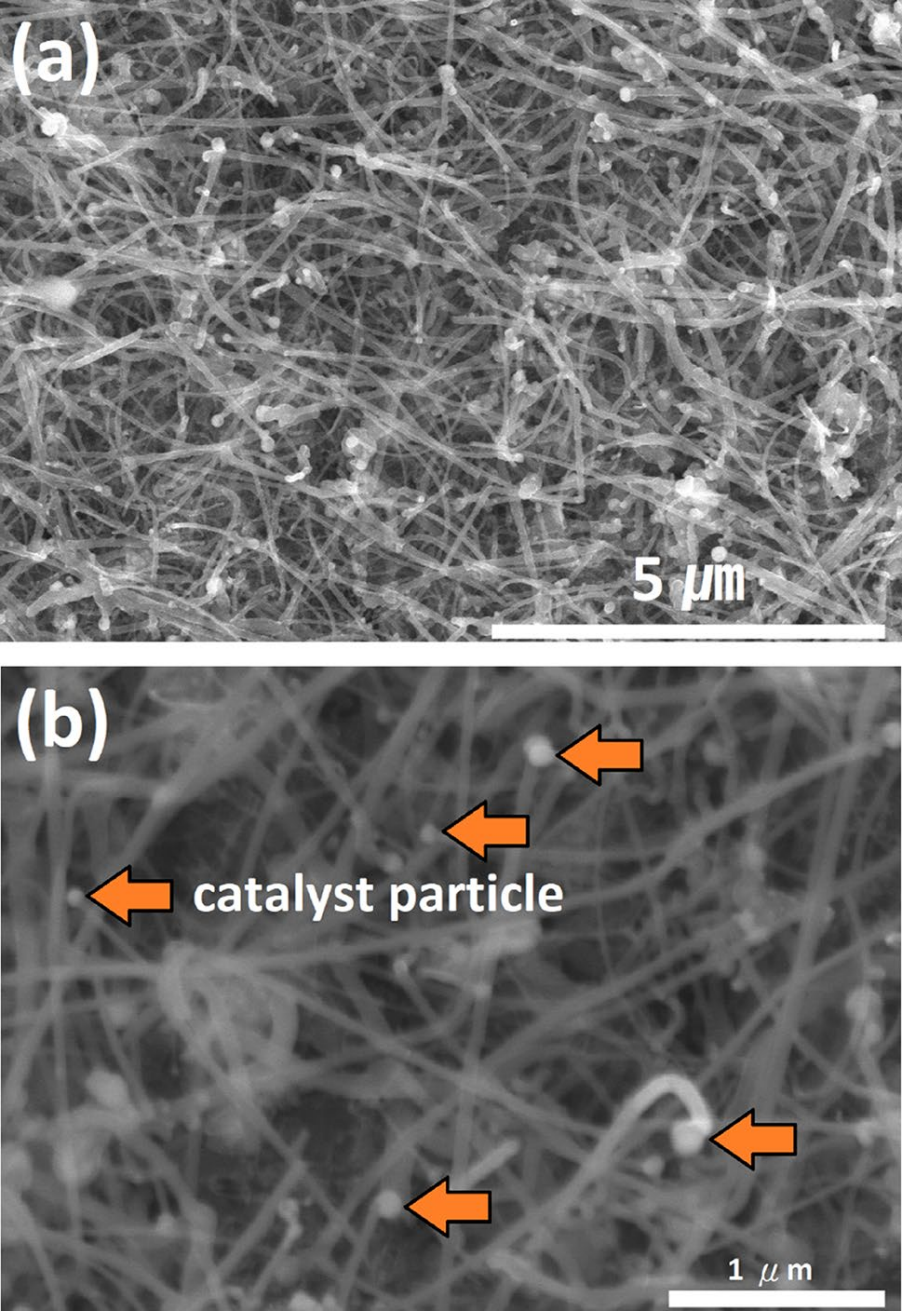
(**a**) Low magnification SEM images of as-synthesized nanowires, (**b**) high magnification SEM images of as-synthesized nanowires.

First, the SEM was used to confirm whether nanowires were generated or not. Figure 1a shows a low-magnification SEM image. It can be seen that silicon carbide nanowires with the length about 10 µm are randomly and densely grown on the substrate. Figure 1b shows a high-magnification SEM image, which clearly reveal the catalyst particles attached to the tip of the nanowire (yellow arrow in the Fig. 1b). This phenomenon is very common in vapor–liquid–solid (VLS) mechanism^48^. The Fig. S3 shows the schematic diagram of as-synthesized nanowires on the substrate.

To estimate the density of SiC/SiO_2_ nanowires, the SEM image is divided into 48 parts, as shown in Fig. S4, and 10 of the 48 parts are randomly selected to count the number of nanowires and ensure that there is no double-counting in the 10 randomly selected parts, then the average of these 10 randomly selected parts is used to estimate the nanowires density in the SEM image and its density is estimated to be 11.81 number/μm^2^, as shown in Table S3.

**Figure 2:**
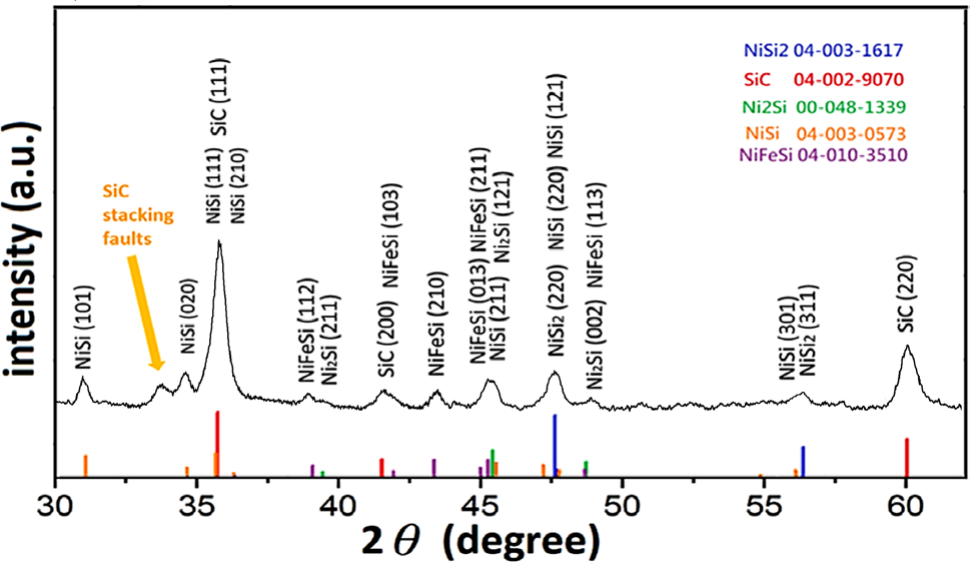
The XRD pattern of as-synthesized nanowires.

Then, the XRD was used to determine the crystalline phases. Figure 2 shows the XRD pattern of as-synthesized SiC/SiO_2_ nanowires. It is indicated that the sample is composed of 3C–SiC and metal silicide (NiSi_2_, NiSi, Ni_2_Si, NiFeSi). The diffraction peaks at 35.82°, 41.60° and 59.95° are aligned with the (111), (200), (220) planes respectively of 3C–SiC (JCPDS: 04-002-9070).

According to the previous reports^45,49^, the minor peak at 33.75° is caused by stacking faults in the (111) planes of 3C–SiC, and the stacking faults density can be evaluated by the intensity ratio (X) of SF peak and SiC (200) peak^50,51^.

1 $$ {\text{X}} = \frac{{I_{SF} }}{{I_{{\left( {200} \right)}} }} $$

where I_SF_ and I_(200)_ are the intensity values of SF peak and SiC (200) peak respectively. A larger ratio value demonstrates the higher stacking faults density in the SiC. However, in order to get more precise value, GSAS-II^52^ software was used to simulate and analyze the effect of stacking faults on XRD of silicon carbide. The results were shown in the Figs. S5 and S6. From the SiC stacking faults XRD simulation (Fig. S6), it can be found that as the stacking faults density increases, the intensity ratio (X) also increases, which corresponds to Eq. (1).

In our experiment (Fig. 2), the intensity ratio ($\frac{{I_{SF} }}{{I_{{\left( {200} \right)}} }}$) is 1.28, which confirms to the simulation result with stacking faults percentage of 19%. It means that the SiC nanowires in this experiment possess 19% stacking faults.

**Figure 3:**
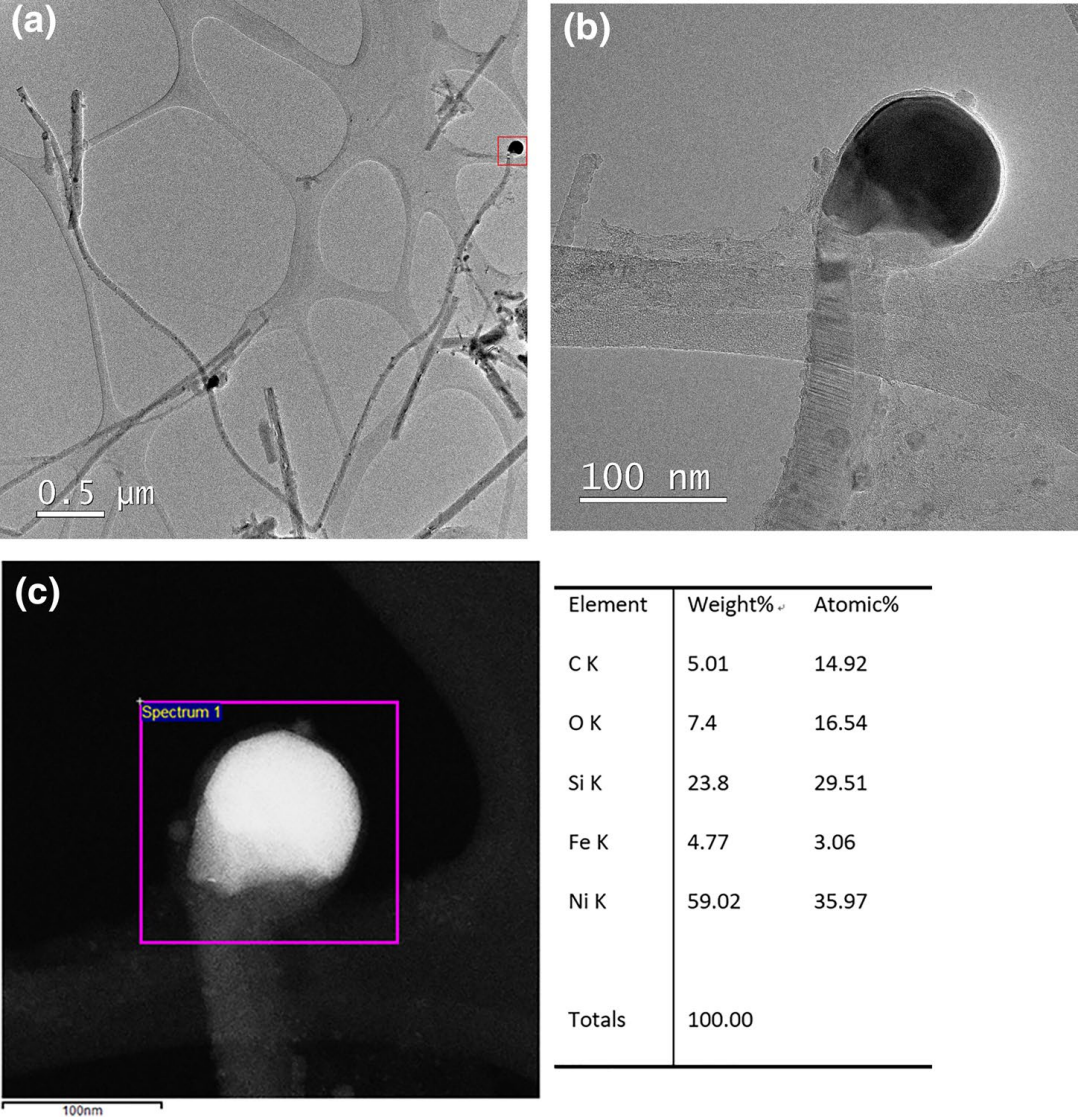
(**a**) The low-magnification TEM image of as-synthesized nanowires. (**b**) The high-magnification TEM image of the selected area marked by the red rectangle in (**a**). (**c**) The EDS pattern of the catalyst particles.

In order to obtain the microstructure and composition of as-synthesized nanowires, TEM and EDS analyses were carried out. Figure 3a is a low-magnification TEM image of as-synthesized nanowires. It can be seen that some nanowires have no catalyst particles at the tip and the length becomes shorter. This is because some nanowires broke and catalyst particles were dropped off during the TEM sample preparation process. Figure 3b is the high magnification TEM of the red rectangle in Fig. 3a. In order to study the composition of the catalyst particles, EDS was performed, and the results are shown in Fig. 3c. The catalyst particles are composed of C, Si, O, Fe, Ni.

**Figure 4:**
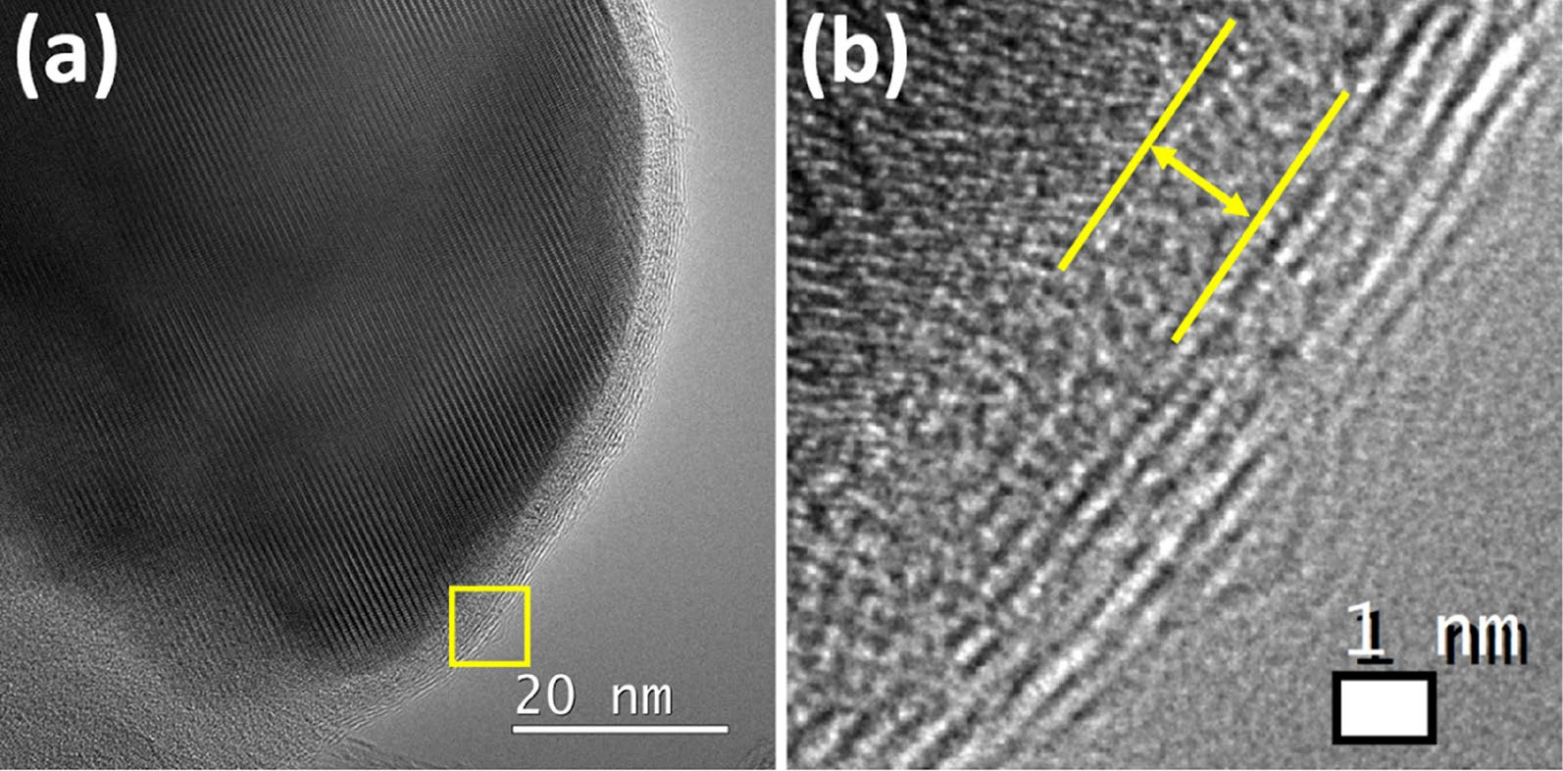
(**a**) The HRTEM image of the catalyst particle (**b**) the partial enlarged image of the selected area marked by the yellow rectangle in (**a**), showing an amorphous layer (SiO_2_) and a multilayered structure (graphene).

Figure 4a is the HRTEM image of the catalyst particles. It can be found that the catalyst particles have good crystallinity and are wrapped by an amorphous layer and a multilayered structure respectively, as shown in Fig. 4b. From the EDS analysis, it can be found out that oxygen is present. So the amorphous layer here could be due to SiO_2_.

**Figure 5:**
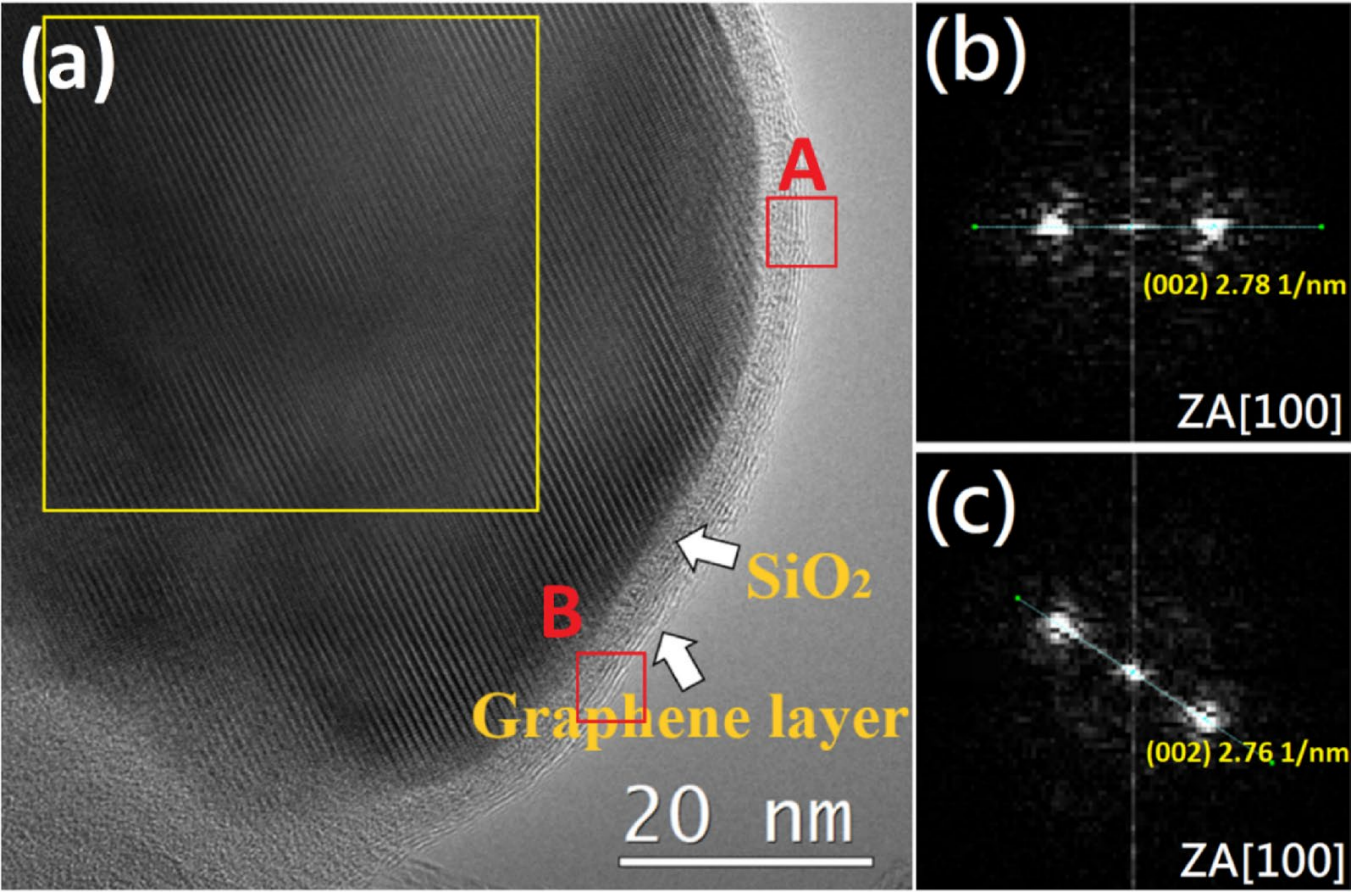
(**a**) The HRTEM image of the catalyst particles. (**b**,**c**) The selected area electron diffraction (SAED) images of the selected area marked by the red rectangle A and B respectively in (**a**).

Next, in order to study the multilayered structure, the selected area electron diffraction (SAED) of red rectangle A and red rectangle B in Fig. 5a was carried out, as shown in Fig. 5b and c. They are found to be consistent with the zone axis [100] of graphene structure (JCPDS card 00-056-0159). Thus, it can be known that the catalyst particles are wrapped by an amorphous SiO_2_ and 5–6 layers of graphene structure respectively.

Figure S7a show the SAED of the catalyst particle structure. It is indexed to the [110] zone axis of Ni_2_Si (JCPDS 00-048-1339) with double diffraction. The diffraction pattern caused by double diffraction was pointed out with yellow arrows in Fig. S7b. The appearance of double diffraction is due to the tip structure of nanowires which is about 100 nm in thickness and is enough to cause double diffraction. Moreover, Fig. S8a shows the STEM image. The STEM images clearly show the position of the atoms, the Ni atoms are in a group of four, showing a staggered arrangement. The position of atoms is consistent with the result of the [110] zone axis of Ni_2_Si (JCPDS 00-048-1339) simulated by JEMS software, as shown in Fig. S8b. However, there is Fe in the analysis of EDS. The size of Fe and Ni atoms are similar, which will replace the part of Ni in Ni_2_Si to form Fe_x_Ni_2−x_Si substitutional solid solution, but its crystal system still belongs to orthorhombic, which can also be obtained from JCPDS card. For example, it might be Fe_0.57_Ni_1.43_ Si (04-017-4935), or FeNiSi (04-010-3510), etc.

**Figure 6:**
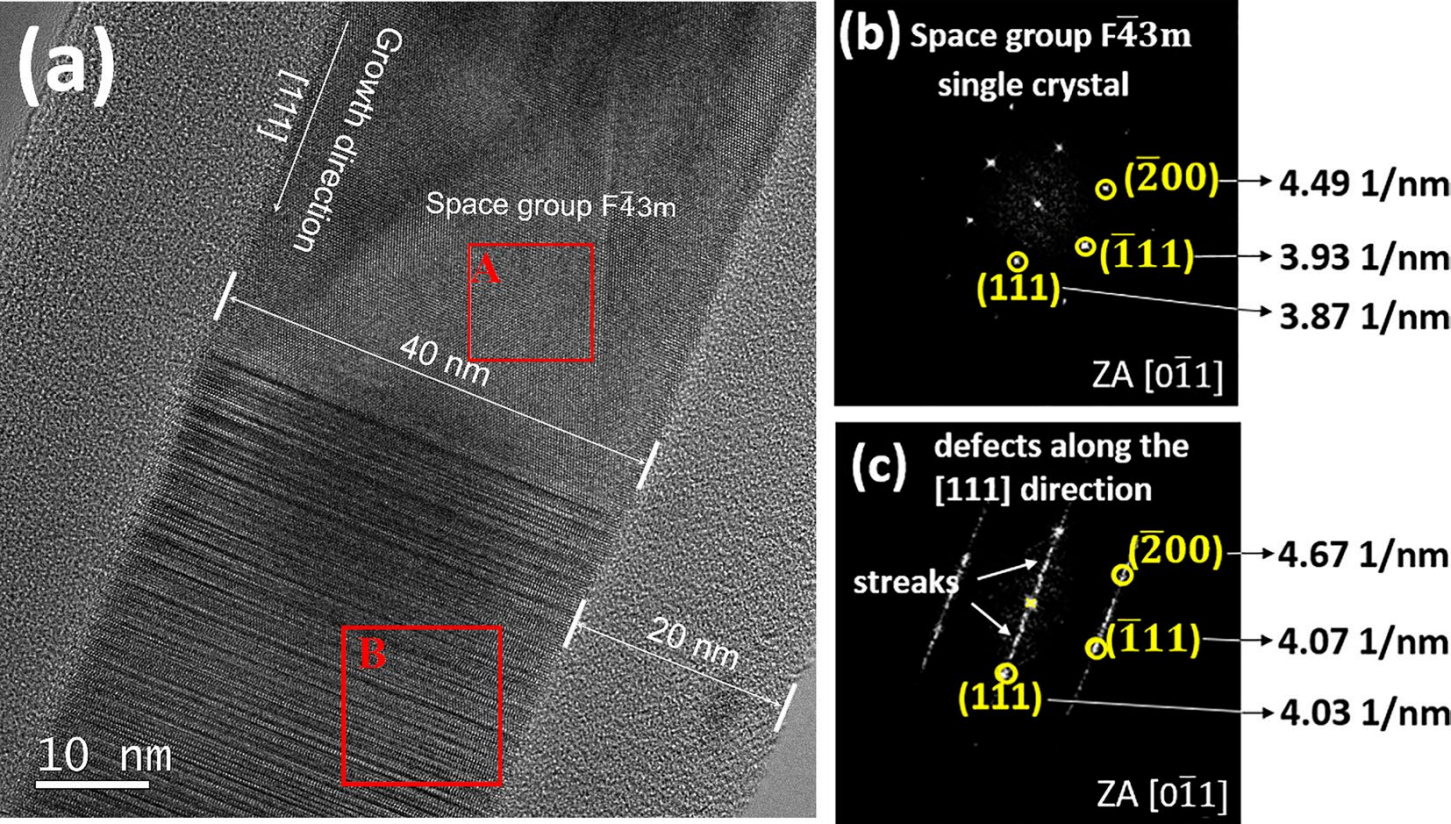
(**a**) The HRTEM image of the nanowire, (**b**) the SAED image of the selected area marked by the red rectangle A in (**a**), showing it consists of a single-crystal structure, (**c**) the SAED image of the selected area marked by the red rectangle B in (**a**), showing the streaks along [111] direction.

Figure 6a shows the HRTEM image of the nanowire and it indicates that the SiC nanowire is covered by 20 nm amorphous SiO_2_ layer, the diameter of SiC nanowire is about 40 nm. Compared with the catalyst particle structure of nanowire, there is no graphene structure found here. Moreover, an obvious difference can be observed in Fig. 6b and c. In Fig. 6b, the diffraction points are spots, which are confirmed to the zone axis [0-11] of 3C–SiC (JCPDS 04-002-9070) simulated by JEMS software, and the diffraction spots are correspond to (111), (− 111), (− 200), respectively. It indicates that the nanowire is a single crystal structure and grows along the [111] direction. However, the diffraction pattern in Fig. 6c shows streaks along the [111] direction, which represents the presence of defects along the [111] direction. To investigate the defect structure of the Fig. 6c in detail, STEM characterization studies were conducted. In Fig. S9, the STEM image shows the planar defects: stacking faults and twins along [111] directions which indicates that the stacking faults exists in (111) plane. The results are consistent with the SAED observations, the streaks along the [111] direction in the SAED is due to the dense stacking faults. From the above analysis, the SiC nanowires consists of single crystal 3C structures (Part A) as well as 3C structures with defects along [111] direction (Part B).

**Figure 7:**
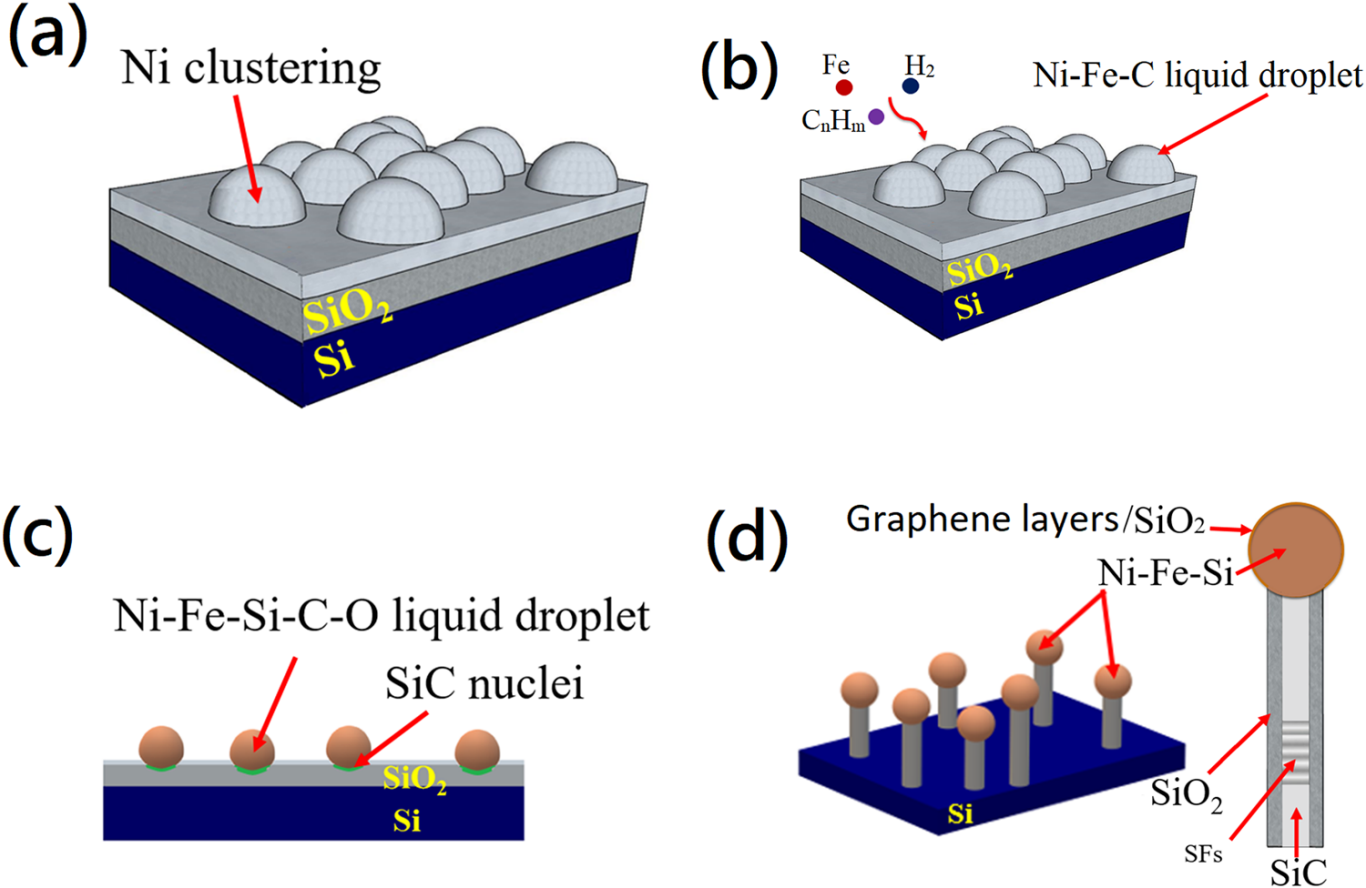
Four steps growth model of SiC/SiO_2_ core–shell nanowires. (**a**) dewetting of the Ni thin film, (**b**) formation of a Ni–Fe–C liquid droplet, (**c**) the nucleation of SiC, (**d**) the growth of SiC/SiO_2_ nanowires.

From the above-mentioned analysis, an approximate model was proposed in Fig. 7 to explain the possible growth mechanism of the SiC/SiO_2_ nanowires.

Based on the existence of catalytic particle on the tips of SiC/SiO_2_ nanowires, the growth process follows the VLS mechanism^48^

1. Catalyst droplet surface adsorbs reactant gas
2. The decomposed species dissolve in the droplet surface
3. The species diffuse into the droplets
4. Precipitation and growth occur at the liquid–solid interface

In this model, the growth of SiC/SiO_2_ nanowires belong to tip-growth and the nanowires growth and the process is divided into four main steps.

### Step (a)

As the temperature increases, Ni film does not react with SiO_2_ layer, but disperses and aggregates into the individual islands on the top of SiO_2_ layer, as shown in Fig. S10.

### Step (b)

As the temperature increases, the ferrocene will sublimate and evaporate then diffuse into the second and third parts of furnace by the N_2_ flow.

When the temperature rise above 500 °C, ferrocene decomposes completely according to the Eq. (2)^45^

2 $$ {\text{Fe}}\left( {{\text{C}}_{5} {\text{H}}_{5} } \right)_{2} \to {\text{Fe}} + {\text{H}}_{2} + {\text{CH}}_{4} + {\text{C}}_{5} {\text{H}}_{6} + {\text{as well as reactive hydrocarbons}} $$

The hydrocarbon gas was adsorbed on the surface of catalyst particles, which decomposed into the carbon atoms and H_2_^29–32^

3 $$ {\text{C}}_{{\text{n}}} {\text{H}}_{{\text{m}}} \to {\text{nC }} + \frac{{\text{m}}}{2}{\text{H}}_{2} $$

Although the growth temperature is 1000 °C, the melting point of the particles will decrease at nanoscale^52^ and the reaction between hydrocarbon gas and Ni is exothermic^53,54^. Thus, the particles will become liquid droplets. The C and Fe will dissolve in the Ni droplet surface and diffuse into it to form Ni–Fe–C liquid droplet.

The Ni catalyst is very important in the process of preparing SiC nanowires at low temperature using the CVD method. Because it can decrease the decomposition temperature of the hydrocarbon gas ^29–32^ and according to the Lim et al. study^44^, the production of CNTs with Ferrocene precursor shows low quality, but the growth with nickel added shows good quality. Thus, the nickel catalyst added can also promote the growth quality of SiC nanowires.

### Step (c)

When the C atoms reach the interface of droplet/SiO_2_, the carbothermal reaction of SiO_2_ layer will be occurred and form SiC^55–57^.

4 $$ {\text{C}} + {\text{SiO}}_{2} \to {\text{SiO}} + {\text{CO}} $$

5 $$ 2{\text{C}} + {\text{SiO}} \to {\text{SiC}} + {\text{CO}} $$

6 $$ {\text{SiO}} + 3{\text{CO}} \to {\text{SiC}} + {\text{CO}}_{2} $$

In addition, during the growth process, the Fe and Si atoms will diffuse into Ni droplet from Ni–Fe–C–Si–O liquid droplet.

### Step (d)

When the SiO_2_ under catalyst particles is completely decomposed, the growth of SiC nanowire will be changed to

7 $$ {\text{C}} + {\text{Si}} \to {\text{SiC,}} $$

the Si atom is provided by the Si substrate.

Meanwhile, the catalyst particles start to lift off due to weak interaction with the substrate.

From the thermodynamic point of view, the reaction (8) can take place during the cooling stage preferably^38^.

8 $$ 3{\text{SiO}}\left( {\text{g}} \right) + {\text{CO}}\left( {\text{g}} \right) \to {\text{SiC}}\left( {\text{s}} \right) + 2{\text{SiO}}_{2} \left( {\text{s}} \right) $$

Thus, the SiO_2_ can wrap on the surface of crystallized SiC nanowires.

Finally, it can be observed that a nanowire consists of a SiC core and SiO_2_ shell with Fe_x_Ni_2−x_Si nanoparticle at the tip.

**Figure 8:**
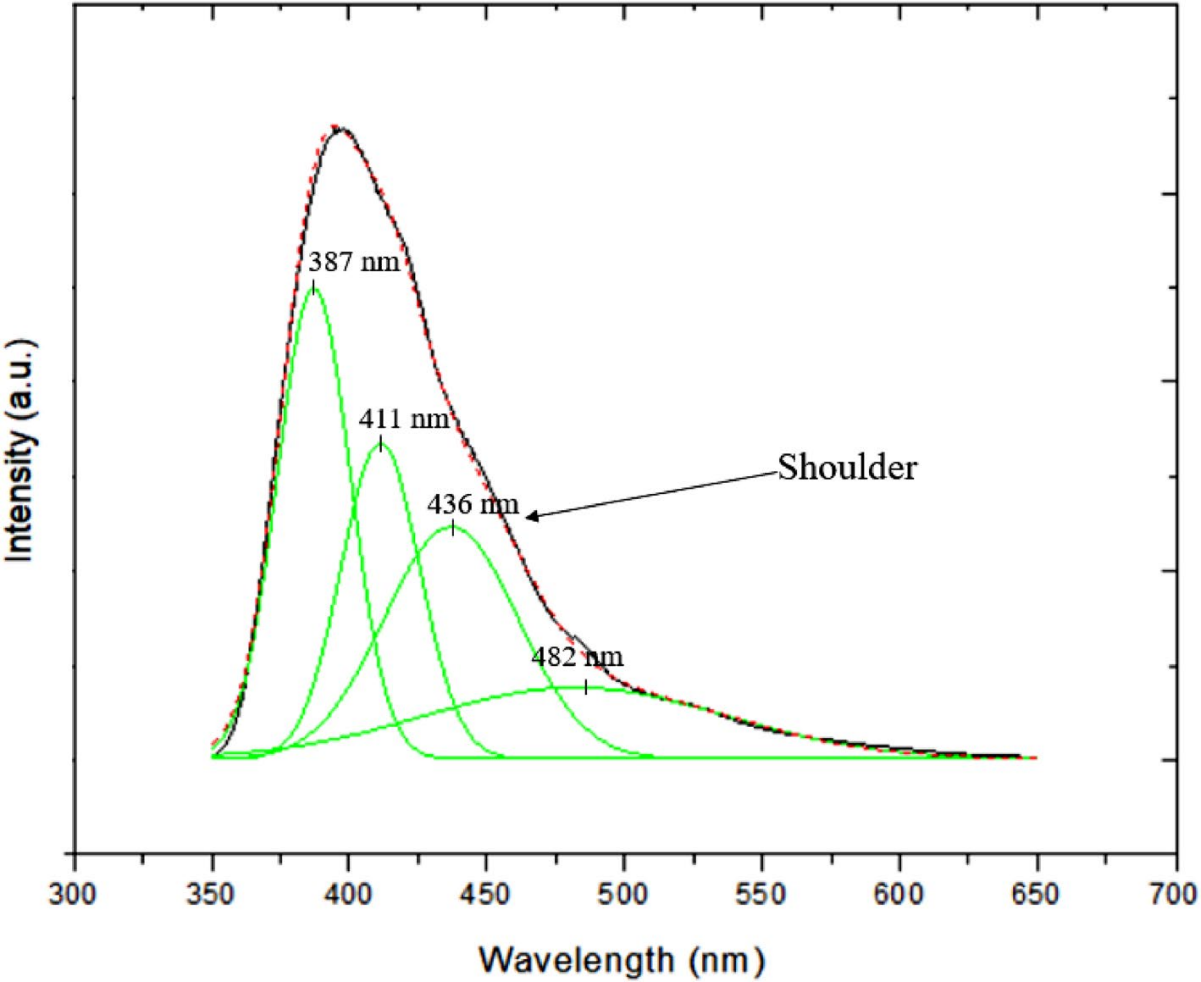
The room-temperature PL spectrum of as-synthesized SiC/SiO_2_ nanowires with the excitation wavelength of 325 nm. The red line is the sum of all decomposition peaks, which fits original experiment data very well.

Here, the SiC/SiO_2_ nanowires were successfully synthesized at lower temperature. In order to study its efficiency in potential emitting device applications, the PL measurement was performed. Figure 8 shows a room-temperature PL spectrum of as-synthesized SiC/SiO_2_ nanowires with the excitation wavelength of 325 nm. As shown in Fig. 8, this spectrum exhibits a strong emission peak at 397 nm (3.12 eV) with a full width at half maximum of 69 nm. Compared with the bandgap of 2.39 eV (525 nm) for bulk 3C–SiC, the silicon carbide nanowires have an obvious blue shift phenomenon. According to the previous reports^9,58–60^, the phenomenon of blue shift can be attributed to the effects of confinement effect and defects (stacking fault) in the silicon carbide nanowires. According to the quantum confinement effect, when the diameter ‘d’ (d = 2r) of the material gradually decreases, the energy gap will increase which causes the phenomenon of blue shift^58^.

9 $$ {\text{E}}^{*} = {\text{E}}_{{\text{g}}} + \frac{{{\text{h}}^{2} }}{{8\upmu {\text{r}}^{2} }} - \frac{{1.8{\text{e}}^{2} }}{{4\uppi \upvarepsilon _{0}\upvarepsilon {\text{r}}}} $$

where r = 1/2d, E_g_ is the bulk 3C–SiC bandgap (2.39 eV), $$\upvarepsilon _{0}$$ = 8.854187817 × $$10^{ - 12} $$F/m, ε ≈ 10 is the vacuum dielectric constant and high frequency dielectric constant respectively. μ is the reduced mass of the exciton, 1/μ = 1/$${\text{m}}_{e}$$ + 1/$${\text{m}}_{h}$$; $${\text{m}}_{e} {\text{ and m}}_{h}$$ is SiC effective mass of electron and effective mass of hole, respectively. Base on the above formula, the 3C–SiC emission peak of the silicon carbide nanowire with a diameter of about 40 nm is about 516 nm, so the phenomenon of blue shift of the emission peak (397 nm) is not due to the quantum confinement effect. The next factor is the defects (stacking fault) in the silicon carbide nanowires. By considering the theory of stacking faults in the silicon carbide nanowires, a spontaneous polarization occurs around each hexagonal turn in 3C–SiC stacking and then create a potential barrier in the conductive band at each hexagonal fault. Finally, it will form quantum well and confine the electrons to the stacking fault layers. Thus, each stacking fault nano-layers may act as SiC polytypism nanosegments^60,61^. In this case, the stacking faults in 3C–SiC produce the 6H–SiC and 4H–SiC nanosegments. Therefore, the structure of the nanowires can be viewed as a 3C–SiC matrix containing 4H and 6H–SiC nanosegments. However, according to the GSAS-II software simulation results, the stacking faults percentage in SiC is only 19%, the emission intensity caused by nano-scale silicon carbide polytypisms should be lower than that of 3C–SiC. However, the PL spectrum (Fig. 8) shows an opposite result. This is due to the size of the nanoscale SiC polytypism is about 1–3 nm. According to Yan et al. study, the indirect bandgap structures will convert into direct bandgap structures when the SiC approach a few nanometers^62^. Besides, the direct bandgap structures will make the higher emission efficiency than that of indirect bandgap structures. This is why the PL spectrum in Fig. 8 shows a strong emission intensity at 397 nm.

Besides, the defects in SiO_2_ can also generate emission peak and can be divided into two types^11,63,64^, one is due to oxygen vacancy around (426–470 nm) and the second is due to twofold coordinated silicon lone-pair centers (–O–Si–O–) around (415–445 nm). The defects in SiO_2_ makes the PL peak asymmetric (the shoulder at the right side in Fig. 8).

According to the above analysis, two methods were used to improve the PL property. First, the diameter of the silicon carbide nanowire was reduced from 40–50 nm to 20–30 nm by changing the thickness of the Ni film on the substrate from 40 to 25 nm. The PL spectrum of new sample is shown in Fig. S11 and it can be seen that the intensity of the right side of the PL emission peak decreases. This phenomenon was also mentioned in the report of Min Liu and Ke-Fu Yao^7^.

Second, the new sample (SiC nanowire diameter: 20 nm) was put into a Teflon beaker which contains 1% HF aqueous solution and performed etching for 3 min and 5 min to remove SiO_2_. After etching, the thickness of the SiO_2_ was successfully decreased and the FWHM is reduced to 60 nm and 56 nm, respectively, as shown in Figs. S12 (3 min), S13 (5 min) and S14 (PL spectrums). These results are consistent with the results of H.T. Liu et al.^11^, the defects in SiO_2_ will produce an emission peak around 426 nm, and the removal of SiO_2_ will decrease the emission intensity of the defects in SiO_2_. The Fig. S15 shows the PL spectrum compared with the previous studies. It can be found that the SiC/SiO_2_ in this study show a good photoluminescent property. All the decomposition peaks in Fig. 8 with different sources are listed in Table 1.

**Table 1:** The sources of decomposition peaks.

Wavelength (nm)	Source
387	SiC nanowires^59^
411	–O–Si–O–^11^
436	O-vacancy^63,64^
482	Bulk 3C–SiC^9^

## Conclusions

In this study, the nickel thin film will be deposited on the substrate in advance. It acts as a catalyst to decrease decomposition temperature of the hydrocarbon gas and promote the growth quality of SiC nanowires. Thus, compared with the previous reports of synthesizing SiC nanowires by ferrocene precursor^37–41^, the growth temperature in our experiment can be reduced to 1000 °C, but the SiC/SiO_2_ nanowires are still successfully and densely synthesized with the lengths about ten micrometers.

In addition, the as-synthesized SiC/SiO_2_ nanowires emit a violet light with high color purity. The reasons for the blue shift phenomenon in the PL spectrum were discussed in detail. The results indicated that the major reason is the stacking faults in 3C–SiC, which results in the formation of nano-scale silicon carbide polytypisms. Unlike bulk SiC polytypisms, the nano-scale SiC polytypisms leads to the band folding, which makes the transition from indirect to direct bandgap and ultimately makes the emission intensity higher. This is why the GSAS-II software simulation results show the faults percentage in SiC is only 19%, but the emission intensity is so strong.

Finally, a possible model was proposed to explain SiC/SiO_2_ nanowires growth mechanism and improve the photoluminescence characteristics of silicon carbide nanowires by changing nickel film thickness and etching.

## Supplementary Information

Supplementary Information.
