# Supplementary material for: Synthesis of SiC/SiO2 core–shell nanowires with good optical properties on Ni/SiO2/Si substrate via ferrocene pyrolysis at low temperature
Source: Sci Rep. 2021 Jan 8;11:233. doi: 10.1038/s41598-020-80580-y (PMC7794414; doi:10.1038/s41598-020-80580-y)
Supplement: Supplementary file 1 — Supplementary Information. [file 41598_2020_80580_MOESM1_ESM.docx]

**SUPPORTING INFORMATION**

**Synthesis of SiC/SiO_2_ core-shell nanowires with good optical properties on Ni/SiO_2_/Si substrate via ferrocene pyrolysis at low temperature**

Bo-Yu Chen^1^, Chong-Chi Chi^1^, Wen-Kuang Hsu^1^, Chuenhou(Hao) Ouyang^1^*

*^1^Department of Materials Science and Engineering, National Tsing Hua University, Hsinchu 30013, Taiwan (R.O.C.)*

*Author to whom correspondence should be addressed. Electronic mail: [houyang@mx.nthu.edu.tw](mailto:houyang@mx.nthu.edu.tw)


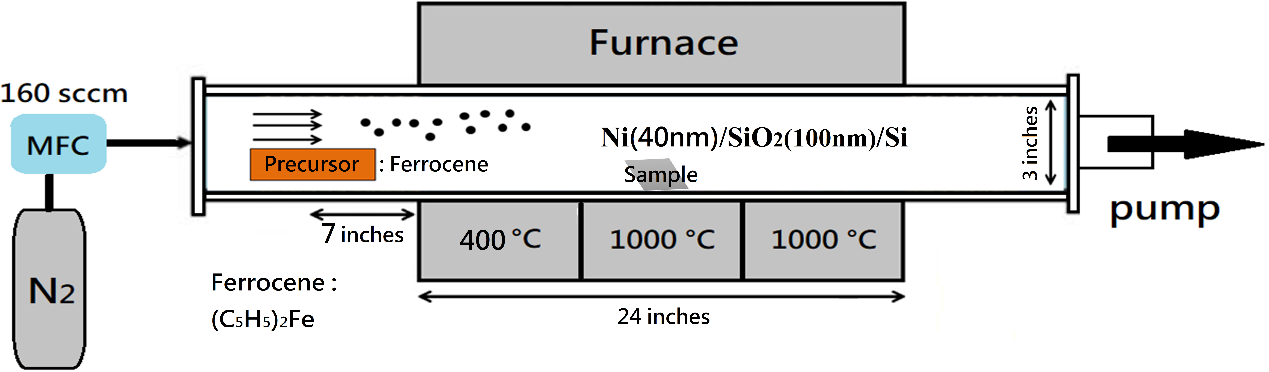


**Fig.S1 Schematic image of the CVD system**

**EXPERIMENT:**

Ni films were deposited on the SiO2(100 nm)/Si substrate with the size 2.0×2.5 cm^2^ at room temperature by ion-beam deposition system under the base pressure of 8.0×10^–8^ Torr in the chamber. Before deposition, the substrate was cleaned by an ultrasonic cleaner for 20 minutes by using acetone, ethanol and DI water and then was dried in nitrogen flow. The Ni target (purity 99.99 %) was pre-sputtered for 10 min to eliminate the contamination on the target surface before deposition. The beam voltage and beam current of the dc ion-source was set to 800 V/7.0 mA and the sputtering pressure is 7.2 × 10^–5^ Torr. The final thickness of Ni film was about 40 nm with deposition rate 0.03 nm/s. After the deposition of Ni, the sample was placed into the quartz tube furnace with a precursor – Ferrocene (C_5_H_5_)_2_Fe (purity 99.0%, 1200 mg), as shown in Fig.S1. The furnace is divided into three parts, where the first part was set to 400 °C, and the second and third parts were adjusted to 1000 °C respectively to grow nanowires. The precursor was placed 7 inches at the left side of the furnace and the sample was put on the second part of the furnace. Before heating, the vacuum in the quartz tube will be evacuated to 1.0×10^–2^ Torr, and then N2 gas (purity 99.999 %) is injected into the quartz tube to 1 atm, and then the vacuum in the quartz tube will be evacuated to 1.0×10^–2^ Torr again, the above steps will be repeated 3 times to purify the atmosphere in the quartz tube. After that, it will take 3 hours to slowly increase the temperature to the target temperature (400 °C, 1000°C, 1000 °C) and then held for 1 hour at a constant N2 flow of 160 SCCM to keep the pressure at 1 atm to grow nanowires. The quartz tube was naturally cooled to room temperature. The parameters of film deposition and CVD were summarized in Table.S1 and Table.S2.

X-ray diffractometer (XRD (Rigaku-TTRAX Ⅲ)) with the Cu target (λ =1.5406 Å) was used to determine the crystalline phases. The scanning electron microscopy (SEM) (FESEM-8000) was used to analyze the surface morphology. The microstructure and composition of the sample were investigated by transmission electron microscopy (TEM) (JEOL ARM-200FTH) with Energy-dispersive X-ray spectroscopy (EDS) and simulation software JEMS (Java Electron Microscopy Simulation Software). For TEM observation, the as-synthesized SiC/SiO_2_ nanowires were dispersed in ethanol (purity 99.5 %)by ultrasonication for 15 min, and then a droplet of solution was dropped onto a lacey carbon-coated copper grid (300 mesh). The detail procedure is shown in Fig.S2. The photoluminescence (PL) spectrum was performed at room temperature using a fluorescence spectrophotometer (PerkinElmer LS55) with the excitation wavelength of 325 nm.

**The preparation of TEM sample**

First, the sample was put into glass bottle containing ethanol (purity 99.5 %), and carefully scraped the sample by flathead screwdriver, as shown in Fig.S2(a), then dispersed by ultrasonication for 15 min. Finally, a droplet of the suspension containing the products was dropped onto a lacey carbon-coated copper grid (300 mesh) and then dried in air at room temperature, as shown in Fig.S2(b).


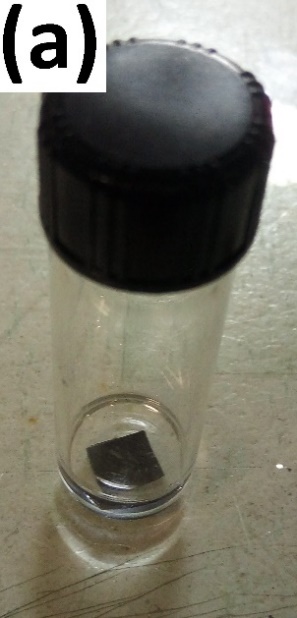

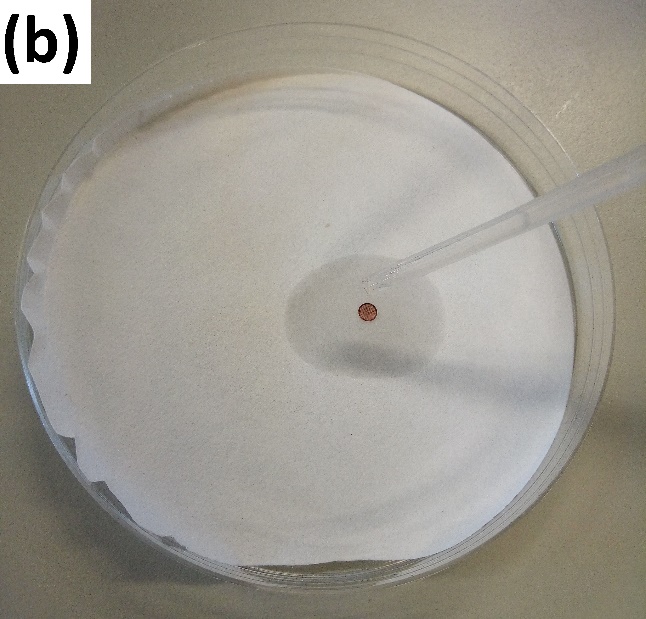


**Fig.S2 (a) a solution of the suspension containing the products, (b) a lacey carbon-coated copper grid with droplets containing product**

**Table.S1 Ion Beam Sputtering Deposition Parameters**

| Base pressure | 8.0×10^-8^ Torr |
| --- | --- |
| Sputtering pressure | 7.2×10^-5^ Torr |
| Substrate size | 2 cm×2.5 cm |
| Beam voltage/current | 800 V, 7.0 mA |
| Film thickness | ~ 40 nm |
| Deposition rate | 0.03 nm/s |

**Table.S2 Furnace Tube Parameters**

| Base pressure | 1.0×10^-2^ Torr |
| --- | --- |
| Working pressure | 1.0 atm |
| Working gas/flow | N_2_, 160 sccm |
| First part temperature | 400 °C |
| Second part temperature | 1000 °C |
| Third part temperature | 1000 °C |


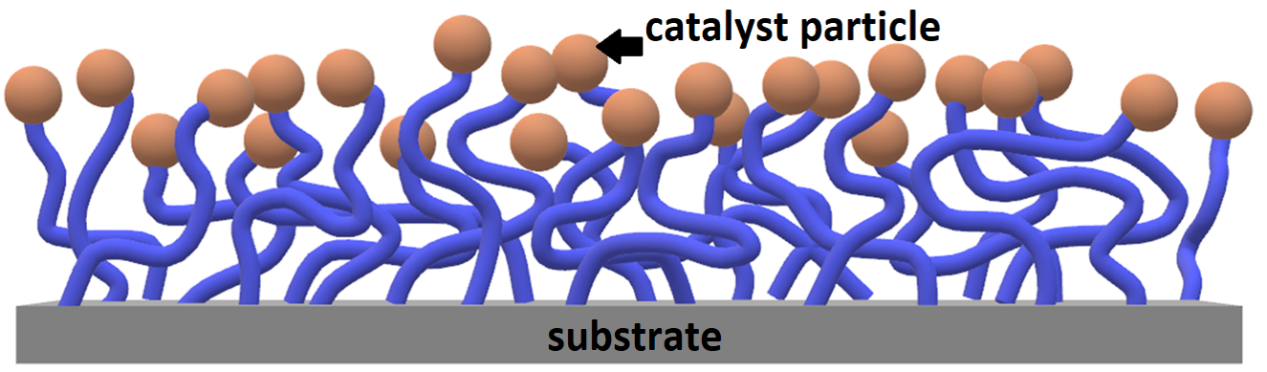


**Fig.S3 the schematic diagram of as-synthesized nanowires on the substrate.**

**Nanowires density**

The SEM image is divided into 48 parts, as shown in Fig.S4, and 10 of the 48 parts are randomly selected to count the number of nanowires and ensure that there is no double-counting in the 10 parts randomly


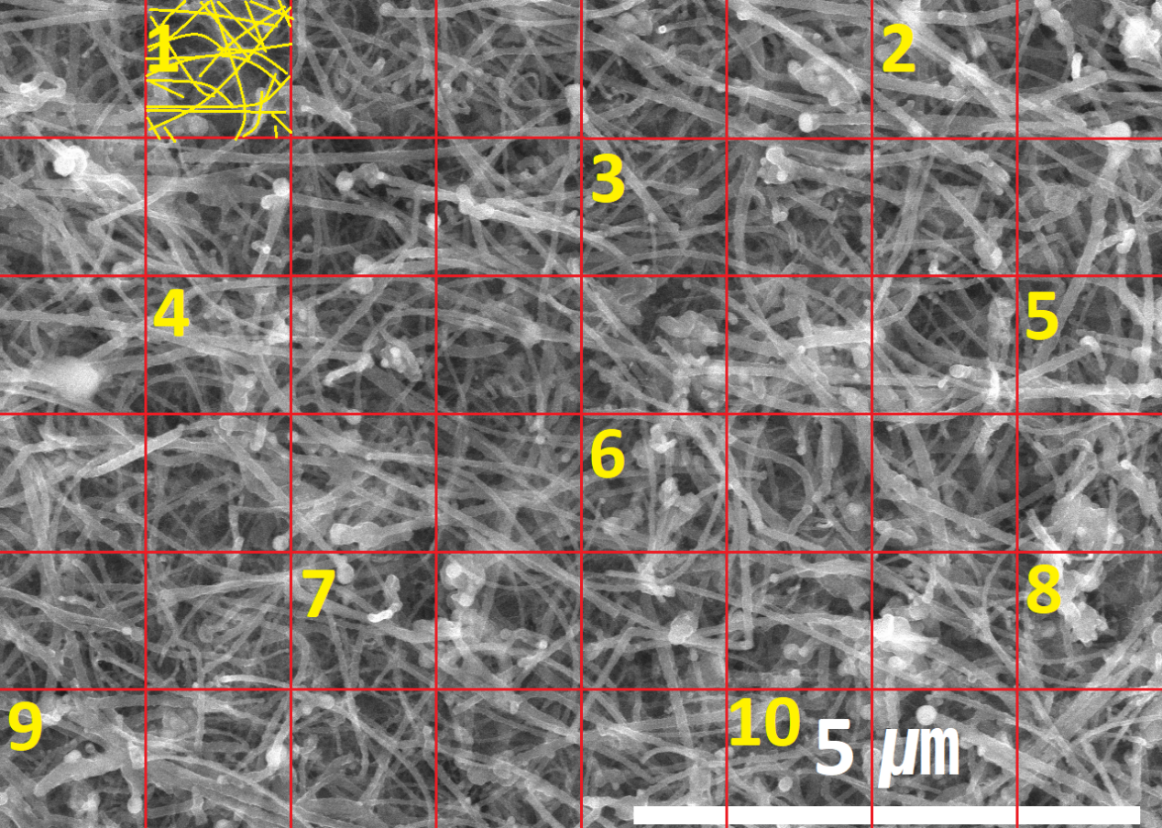


**Fig.S4 the randomly selected parts used to count the number of nanowires in Fig.1(a)**

The 10 randomly selected parts are numbered 1-10 sequentially and ensure that there is no double-counting, as shown in Fig.S4. The area of each part is 1.94 μm^2^ and the number of nanowires for each selected part will be calculated manually. The calculated results are shown in Table. S3.

**Table.S3 The calculated results of the number of nanowires for each selected part**

| Part | number of nanowires | Density (#/μm^2^) | Average density(#/μm^2^) |
| --- | --- | --- | --- |
| 1 | 26 | 13.40 | 11.81 |
| 2 | 22 | 11.34 |  |
| 3 | 23 | 11.86 |  |
| 4 | 29 | 14.95 |  |
| 5 | 18 | 9.28 |  |
| 6 | 27 | 13.92 |  |
| 7 | 25 | 12.89 |  |
| 8 | 18 | 9.28 |  |
| 9 | 21 | 10.82 |  |
| 10 | 20 | 10.31 |  |


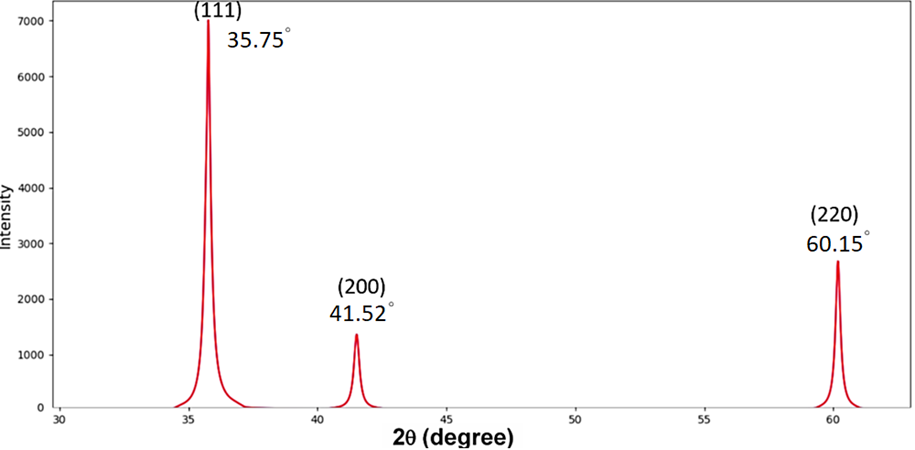


**Fig.S5 the XRD simulation of 3C-SiC without stacking faults by GSAS-II software**


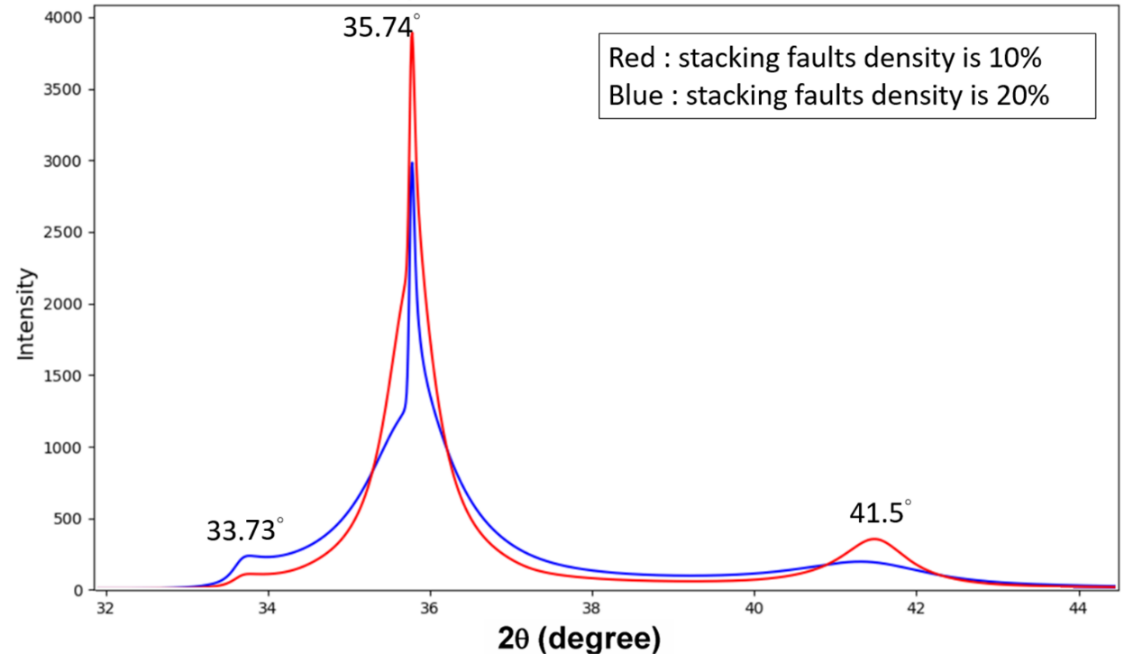


**Fig.S6 the XRD simulation of 3C-SiC with stacking faults by GSAS-II software. (red line: faults density is 10% and blue line faults density is 20%) The diffraction peaks at 35.74° and 41.5° are aligned with the (111), (200) planes respectively of 3C-SiC and the peak at 33.73° is SF peak.**

The Fig.S5 is a XRD simulation of 3C-SiC (no stacking faults). It can be observed that there is no SF diffraction peak on the left side of the 35.75° diffraction peak, and each diffraction peak position and intensity are also corresponds to the 3C-SiC (JCPDS 04-002-9070).

According to the previous reports [1-2], the minor peak at 33.75° is caused by stacking faults in the (111) planes of 3C-SiC, and the stacking faults density can be evaluated by the intensity ratio (X) of SF peak and SiC (200) peak. [3-4]

X = $\frac{I_{SF}}{I_{(200)}}$ ……………………………………..(1)

where I_SF_ and I _(200)_ are the intensity values of SF peak and SiC (200) peak respectively. However, in order to get more precise value, GSAS-II [5] software was used to simulate and analyze the amount of stacking faults on XRD of silicon carbide. The results were shown in the Fig.S6. From the SiC stacking faults XRD simulation (Fig.S6), it can be found that as the stacking faults density increases, the intensity ratio (X) also increases, which corresponds to eq.(1). However, a peak at 33.73° was observed in the presence of stacking faults, as shown in Fig.2, and the simulation results were also similar to those reported in the previous studies. [1-2]


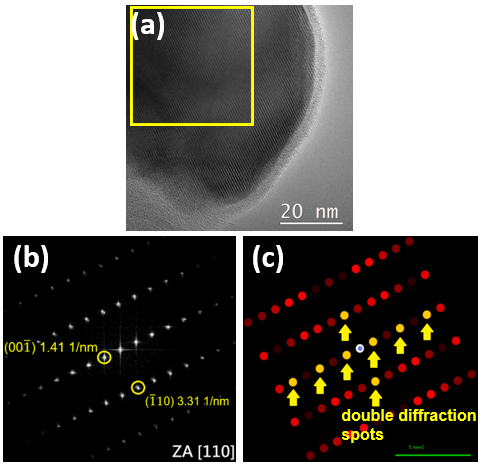


**Fig.S7(a) the HRTEM image of the catalyst particle, (b)** **the selected area electron diffraction (SAED) image of the selected area marked by the yellow rectangle in (a), (c) the SAED (with double diffraction) of the [110] zone axis of Ni_2_Si (JCPDS 00-048-1339) simulated by JEMS software**


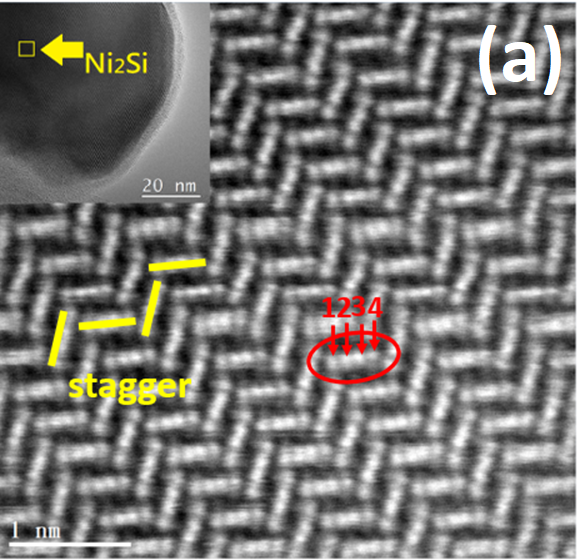

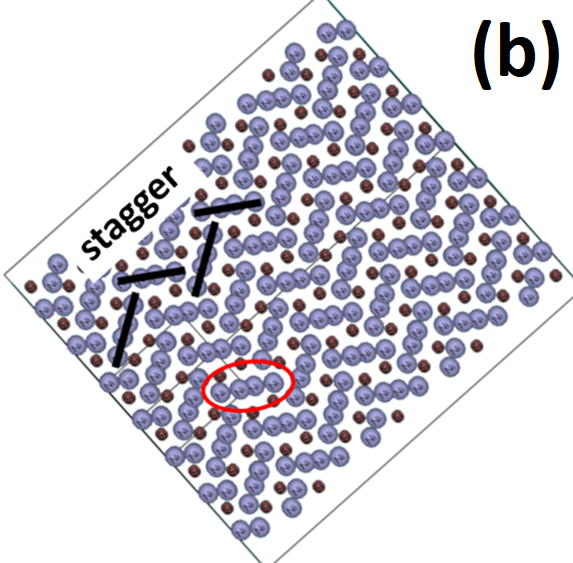


**Fig.S8(a) the STEM image of catalyst particle (b) The atoms position simulated by JEMS software (JCPDS Ni2Si 00-048-1339 with [110] zone axis)**

**
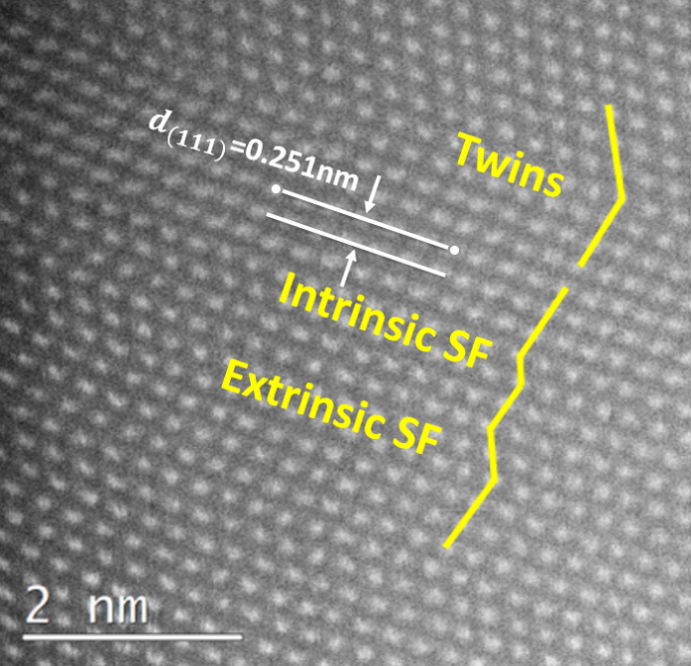
**

**Fig.S9 the STEM image of SiC nanowires in defects sections, showing the intrinsic, extrinsic stacking faults and twins**


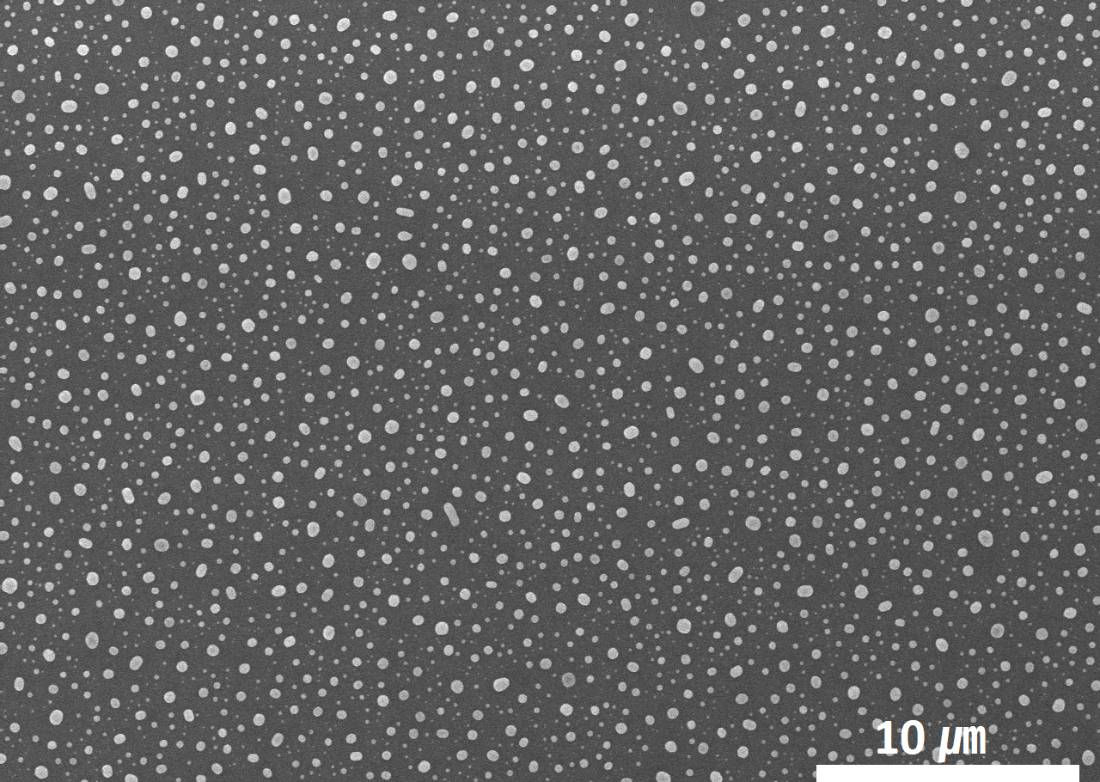


**Fig.S10 the SEM image of Ni/SiO_2_/Si sample annealed at 1000 °C for 1s (heating rate: 5.4 °C/min)**


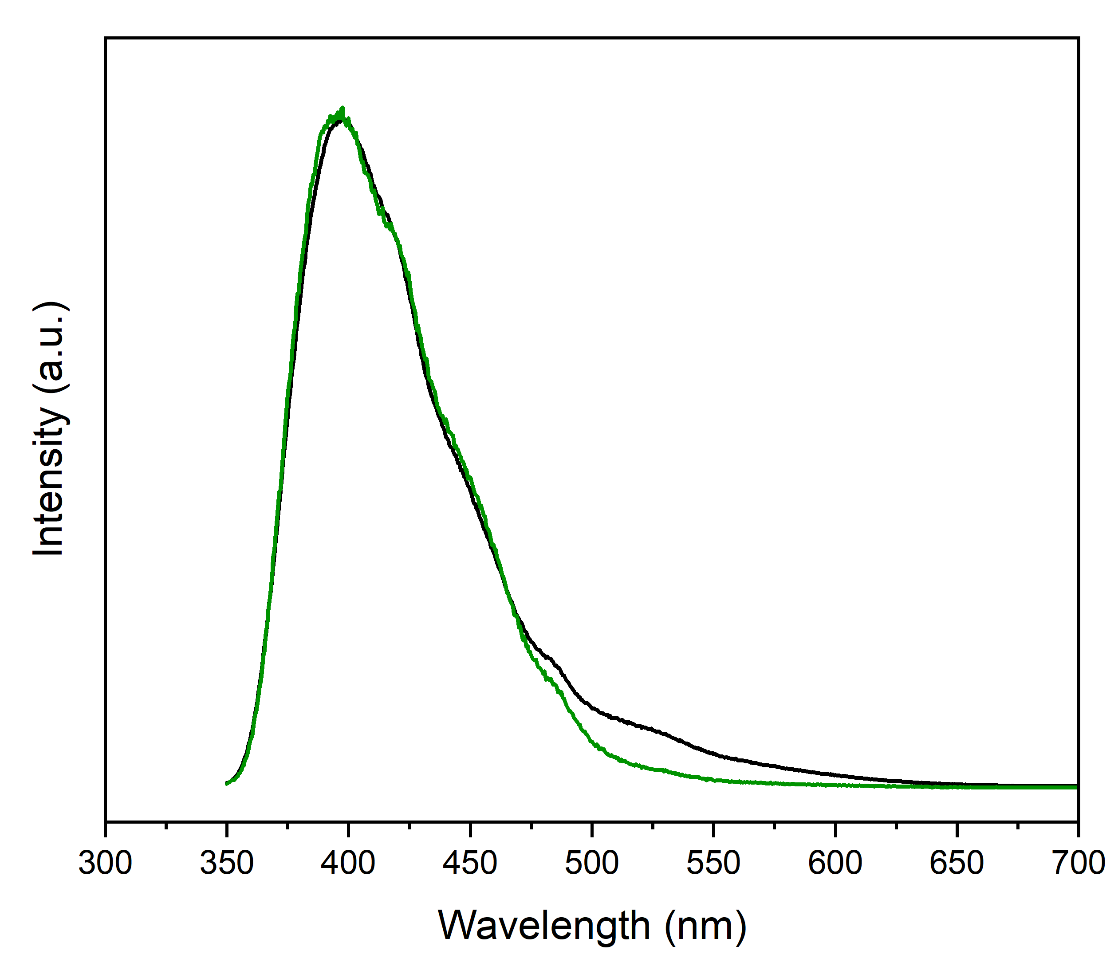


**Fig.S11 the room-temperature PL spectrum of SiC/SiO_2_ nanowires with different** **diameter (black line:40-50 nm, green line: 20-30 nm)**


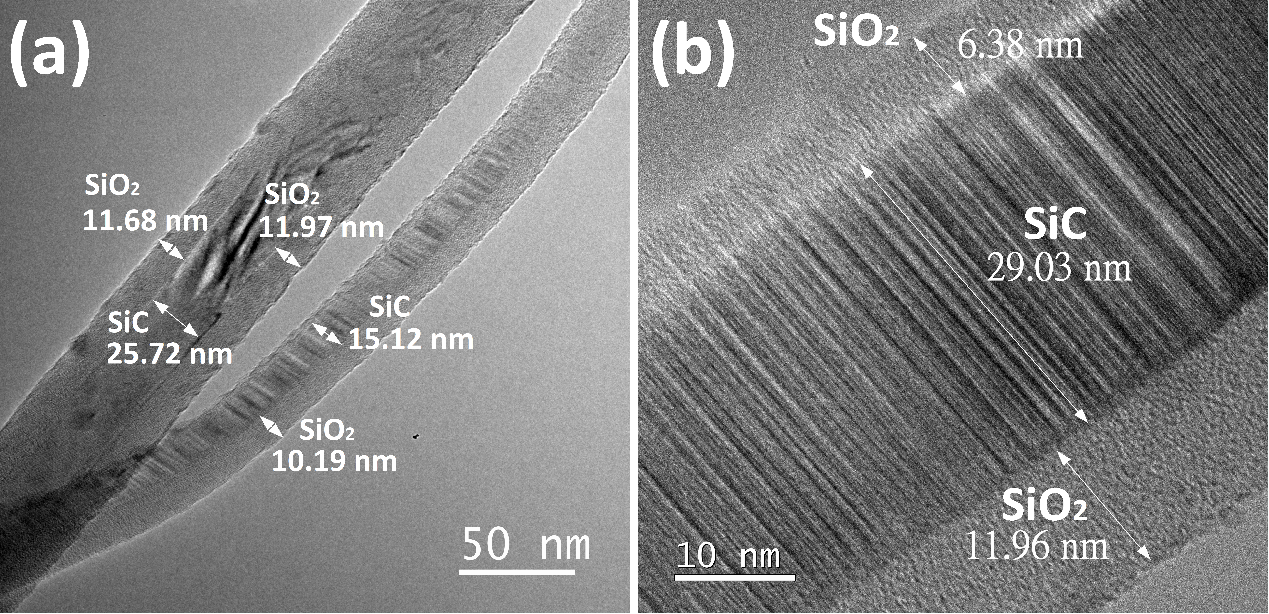


**Fig.S12 (a) the TEM image (b) the HRTEM image of SiC/SiO_2_ nanowires** **etched in 1% HF solution for 3 minutes, showing a thinner (6-12 nm) amorphous SiO_2_ shell**


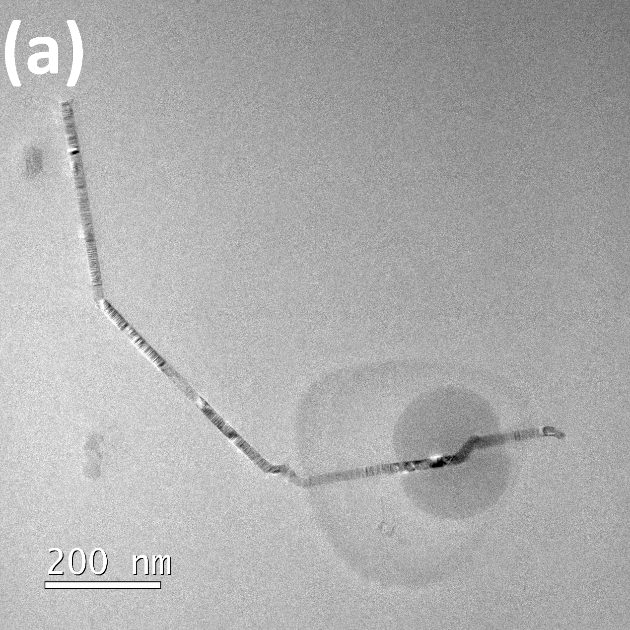

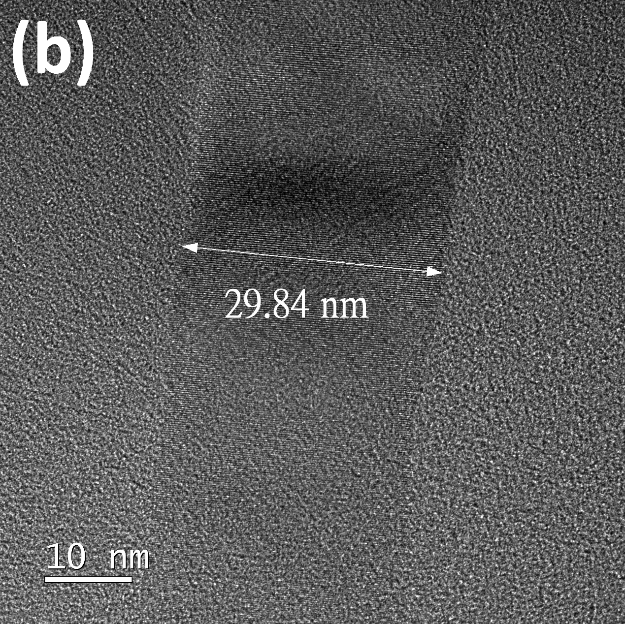


**Fig.S13 (a) the TEM image (b) the HRTEM image of SiC/SiO_2_ nanowires etched in 1% HF solution for 5 minutes, showing the SiC nanowire without SiO_2_ shell**


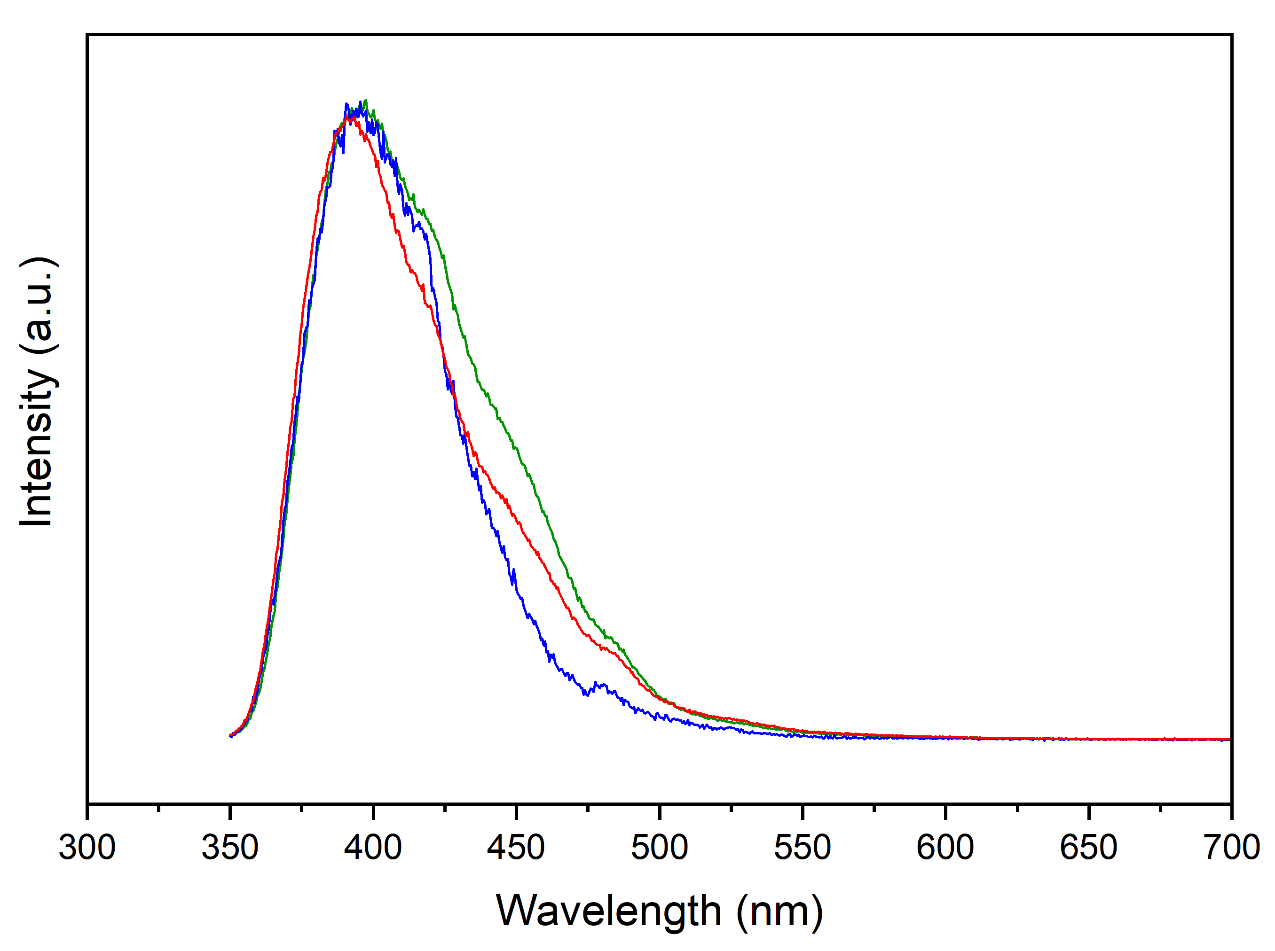


**Fig.S14 the room-temperature** **PL spectrum of SiC/SiO_2_ nanowires with/without etching (green line: before etching, red line: after etching 3 min, blue line: after etching 5 min)**


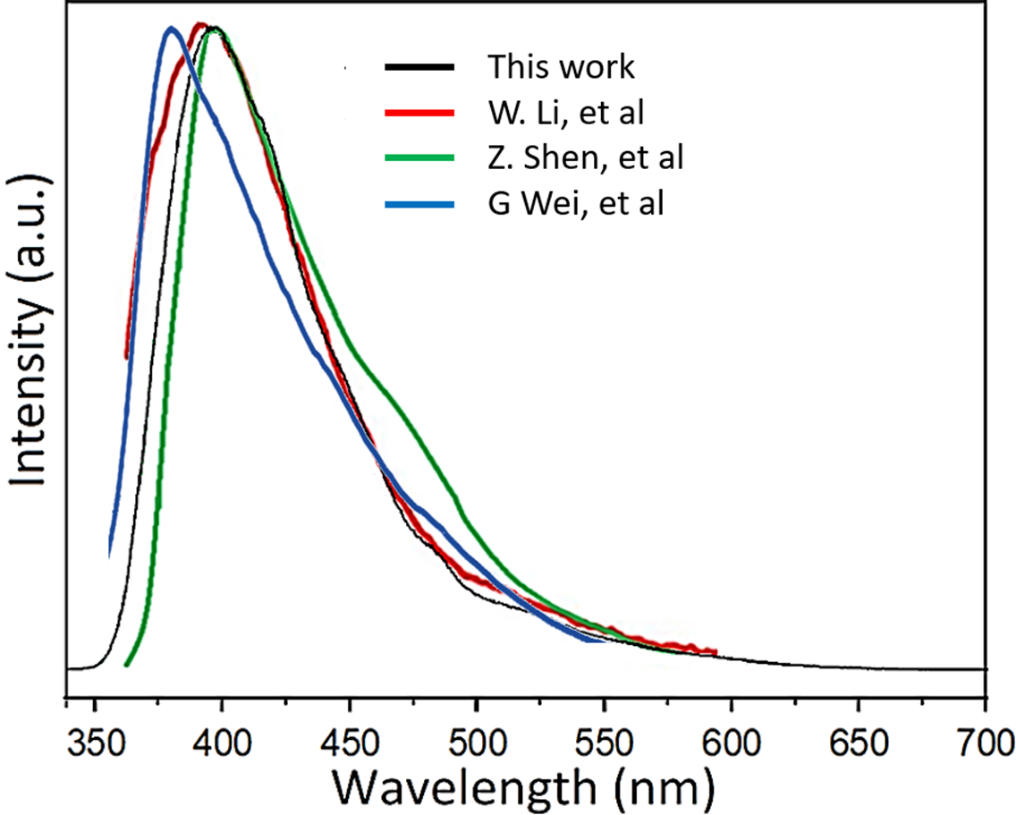


**Fig.S15 the PL spectrum compared with the previous studies. (The FWHM of the red line[6], green line[7] and blue line[8] are 78 nm, 72 nm and 70 nm, respectively)**

**References**

[1] V.V. Pujar, J.D. Cawley, **Effect of stacking faults on the X-ray diffraction profiles of β-SiC powders**, J. Am. Ceram. Soc., 78 (1995), pp. 774-782

[2] V.V. Pujar, J.D. Cawley, **Computer simulations of diffraction effects due to stacking faults in β-SiC: I, simulation results**, J. Am. Ceram. Soc., 80 (2005), pp. 1653-1662

[3] H.Y. Zhang, Y.J. Xu, J.G. Zhou, J.F. Jiao, Y.J. Chen, H. Wang, C.Y. Liu, Z.H. Jiang, Z.J. Wang, **Stacking fault and unoccupied densities of state dependence of electromagnetic wave absorption in SiC nanowires**, J. Mater. Chem. C, 3 (2015), pp. 4416-4423

[4] J. Kuang, W. Cao, **Stacking faults induced high dielectric permittivity of SiC wires**, Appl. Phys. Lett., 103 (11) (2013), pp. 112906

[5] GSAS-II manual. <https://subversion.xray.aps.anl.gov/trac/pyGSAS>

[6] W. Li, Q.L. Jia, X.H. Liu, J. Zhang, **Large scale synthesis and photoluminescence properties of necklace-like SiC/SiOx heterojunctions via a molten salt mediated vapor reaction technique**, Ceram. Int., 43 (2017), pp. 2950-2955

[7] Z. Shen, J. Chen, B. Li, G. Li, J. Li, X. Hou, **A novel two-stage synthesis for 3C–SiC nanowires by carbothermic reduction and their photoluminescence properties**, J. Mater. Sci., 54 (2019), pp. 12450-12462

[8]G.D. Wei, W.P. Qin, R. Kim, G.F. Wang, P.F. Zhu, D.S. Zhang, K.Z. Zheng, L.L. Wang, **Large-scale synthesis and photoluminescence properties of SiC networks,** Appl. Phys. A, 96 (2009), pp. 521-527
